# Radiosensitization Effect of Gold Nanoparticles on Plasmid DNA Damage Induced by Therapeutic MV X-rays

**DOI:** 10.3390/nano12050771

**Published:** 2022-02-25

**Authors:** Katsunori Yogo, Masaki Misawa, Hidetoshi Shimizu, Tomoki Kitagawa, Ryoichi Hirayama, Hiromichi Ishiyama, Hiroshi Yasuda, Satoshi Kametaka, Seiichi Takami

**Affiliations:** 1 Graduate School of Medicine, Nagoya University, 1-1-20 Daiko-minami, Higashi-ku, Nagoya 461-8673, Japan; kametaks@met.nagoya-u.ac.jp; 2 Health and Medical Research Institute, National Institute of Advanced Industrial Science & Technology (AIST), 1-2-1 Namiki, Tsukuba 305-8564, Japan; m.misawa@aist.go.jp; 3 Department of Radiation Oncology, Aichi Cancer Center Hospital, 1-1 Kanokoden, Chikusa-ku, Nagoya 464-8681, Japan; hishimizu@aichi-cc.jp (H.S.); tkitagawa_5410@yahoo.co.jp (T.K.); 4 Institute for Quantum Medical Science (iQMS), National Institutes for Quantum Science and Technology (QST), 4-9-1 Anagawa, Inage-ku, Chiba 263-8555, Japan; hirayama.ryoichi@qst.go.jp; 5 Graduate School of Medical Science, Kitasato University, 1-15-1 Kitazato, Minami-ku, Sagamihara 252-0374, Japan; hishiyam@kitasato-u.ac.jp; 6 Department of Radiation Biophysics, Research Institute for Radiation Biology and Medicine, Hiroshima University, 1-2-3 Kasumi, Minami-ku, Hiroshima 734-8553, Japan; hyasuda@hiroshima-u.ac.jp; 7 Graduate School of Engineering, Nagoya University, Furo-cho, Chikusa-ku, Nagoya 464-8603, Japan; takami.seiichi@material.nagoya-u.ac.jp

**Keywords:** gold nanoparticle, radiation therapy, DNA damage, radiosensitizer, MV X-rays, positively charged nanoparticle

## Abstract

Gold nanoparticles (AuNPs) can be used with megavolt (MV) X-rays to exert radiosensitization effects, as demonstrated in cell survival assays and mouse experiments. However, the detailed mechanisms are not clear; besides physical dose enhancement, several chemical and biological processes have been proposed. Reducing the AuNP concentration while achieving sufficient enhancement is necessary for the clinical application of AuNPs. Here, we used positively charged (+) AuNPs to determine the radiosensitization effects of AuNPs combined with MV X-rays on DNA damage in vitro. We examined the effect of low concentrations of AuNPs on DNA damage and reactive oxygen species (ROS) generation. DNA damage was promoted by 1.4 nm +AuNP with dose enhancement factors of 1.4 ± 0.2 for single-strand breaks and 1.2 ± 0.1 for double-strand breaks. +AuNPs combined with MV X-rays induced radiosensitization at the DNA level, indicating that the effects were physical and/or chemical. Although −AuNPs induced similar ROS levels, they did not cause considerable DNA damage. Thus, dose enhancement by low concentrations of +AuNPs may have occurred with the increase in the local +AuNP concentration around DNA or via DNA binding. +AuNPs showed stronger radiosensitization effects than −AuNPs. Combining +AuNPs with MV X-rays in radiation therapy may improve clinical outcomes.

## 1. Introduction

Radiation therapy, one of the widely used cancer treatment methods, is mainly performed using X-rays in megavoltage (MV) energy ranges [1]. Techniques such as intensity-modulated radiation therapy can deliver high doses of radiation to the tumor while sparing the adjacent normal tissues [1,2]. However, treatment outcomes remain insufficient because the normal tissues around the tumor prevent the maximum dose delivery to tumors. Therefore, advances in dose-enhancement techniques are necessary to improve clinical outcomes. Combining radiation therapy with a dose sensitizer may be effective for treating tumors. Gold nanoparticles (AuNPs) [3,4,5] are relatively safe for use in vivo, and they exhibit easy surface modification compared to other high-atomic-number (Z) materials. Physical dose enhancement using AuNPs for kV X-rays occurs with the increase in photoelectric absorption, which is dependent on Z and proportional to Z^3^–Z^5^ [5], and an increase in reactive oxygen species (ROS) generation [6]. Previous studies on X-rays in the kV energy ranges have shown that AuNPs alter radiosensitization to affect DNA damage, cell survival, and treatment effects in mice [7,8,9].

Radiation therapy is mainly performed using MV X-rays, which can treat tumors deep inside the body, thereby reducing effects on the skin. Monte Carlo (MC) simulation, a theoretical calculation method, is often used to evaluate the physical dose enhancement of AuNPs [10]. Considering only physical dose enhancement, the radiosensitization effect of AuNPs is remarkable at kV but not at MV energy ranges. However, in several previous studies, AuNP radiosensitization using MV X-rays has been achieved both in vitro and in vivo [11,12,13,14,15,16]. Jain et al. reported that AuNPs (1.9 nm, 12 μM) promote toxicity in breast cancer cells using MV X-rays (dose enhancement factor (DEF) = 1.29 for 6 MV and 1.16 for 15 MV X-rays) [11]. Chithrani et al. used HeLa cells with 50 nm AuNPs and reported that the DEF was 1.17 for 6 MV X-rays [12]. Wolfe et al. found that AuNPs targeting prostate cancer cells delayed tumor growth in mice treated with MV photons [14].

The precise mechanisms of AuNP-induced radiosensitization are unclear for MV X-rays. In addition to physical dose enhancement, several biological enhancement processes have been proposed [5], including oxidative stress induced by ROS production and dysfunction of mitochondria, cell cycle effects, and DNA repair inhibition. Mitochondria dysfunction may occur because of the high intracellular ROS levels induced by AuNPs. The cell cycle effect depends on the physical and chemical properties of NPs and cell lines. Currently, DNA repair inhibition is not considered to result in enhancement.

In addition, MC calculations have indicated that a high concentration of AuNPs (2000 mg/g) is necessary to treat tumors with MV X-rays, which is an unrealistic concentration in clinical practice [10]. The lethal dose 50% of AuNPs of ~3.2 g/kg body weight in mice has been reported [3]. Moreover, cell survival assays also require relatively high concentrations of AuNPs [11,12,13,14,15,16]. Thus, the concentration of AuNPs required for significant dose enhancement must be reduced before clinical applications.

Studies using MV X-rays are necessary for the wider application of AuNPs. Radiation is known to injure cancer cells by triggering DNA damage. Therefore, investigating whether AuNPs affect radiation-induced events at the DNA level is important for clinical applications. Furthermore, in vitro analysis at the DNA level may eliminate the contributions of biological effects, such as cell cycle regulation and apoptosis [5]. In this study, we investigated the radiosensitization effects of AuNPs on DNA damage induced by MV X-ray irradiation in vitro. Simple, easy, and highly sensitive plasmid DNA assays were performed to quantify DNA damage induced by radiation [17,18,19,20,21].

**Figure 1:**
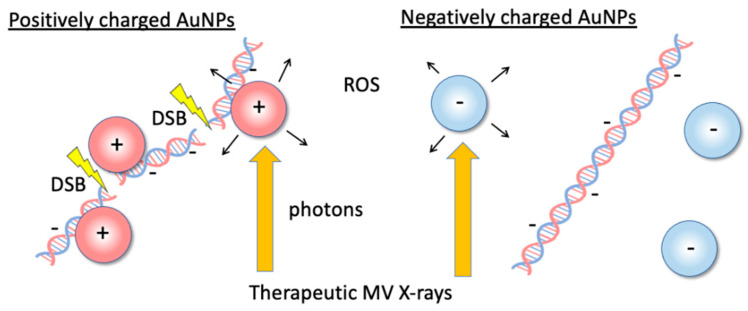
Illustrations of the dose-enhancement mechanism with low concentrations of positively charged gold nanoparticles (+AuNPs) via +AuNP–DNA binding or increasing the local concentration of +AuNPs around DNA. Positively charged +AuNPs used in combination with 6 MV X-rays increased single- and double-strand breaks in plasmid DNA.

In our previous work [22], we propose the use of positively charged (+)AuNPs to reduce AuNP concentrations (Figure 1) and assessed the ROS production in +AuNP and −AuNP solutions to determine the effects of the surface charge of AuNPs for ^192^Ir source gamma-rays (~350 keV) which are widely used for brachytherapy. It is desirable to investigate the applicability of this method to a variety of radiation therapy techniques such as external beam therapy using MV X-rays.

## 2. Materials and Methods

### 2.1. AuNPs and DNA

The 1.4 nm +AuNPs were purchased from Nanoprobes (Cat# 2022, Nanoprobes, Yaphank, NY, USA) [22]. The positive charges were conferred by modifying the AuNP surface with amine groups. We used 2 nm −AuNPs (Cat# EM.GC2, British BioCell International, Cardiff, UK) for comparison. The negative charges developed during nanoparticle synthesis using citrate ligands remained after synthesis. AuNPs were not aggregated.

AuNPs were characterized using scanning transmission electron microscopy (STEM), zeta potential measurement, and dynamic light scattering (DLS) measurement. High-angle annular dark field (HAADF)-STEM images were captured using JEM-ARM 200F (JEOL Ltd., Tokyo, Japan) operated at 200 kV with a thermal field emission gun. The diameter of AuNPs was measured from HAADF-STEM images by analyzing with ImageJ, v.1.47 (NIH). Zeta potential, indicating the surface charge of AuNPs, was measured using ZetaSizer Nano-S (Malvern Instruments, Malvern, UK). DLS measurements were also performed with ZetaSizer Nano-S. We used 640 ng/mL AuNP samples to obtain sufficient DLS signals for 1–2 nm AuNPs. DLS from AuNPs alone and AuNPs with DNA was measured to study the aggregation of AuNPs induced by the addition of DNA.

As the DNA substrate, we used 4.3 kbp pBR322 plasmid DNA (Cat# 319-00444, Nippon Gene, Tokyo, Japan). The plasmid DNA was precipitated using ethanol, resuspended in pure water for buffer exchange [23,24,25], and stored at −20 °C until use. The DNA concentration was quantified by measuring the absorption of the sample at 260 nm using a spectrometer (NanoDrop, Thermo Fisher Scientific, Waltham, MA, USA). We used a molar extinction coefficient of 50 (μg/mL)^−1^ cm^−1^ for double-stranded DNA in this study. DNA solution (500 ng) was prepared in 0.25 mM Tris-Cl buffer (0.25 mM Tris, 0.025 mM ethylenediaminetetraacetic acid (EDTA), pH 7.5). Each Eppendorf tube (0.5 mL) contained 20 μL of irradiated samples. AuNPs were diluted to the desired concentration with 0.25 mM Tris-Cl buffer. The final concentration of AuNP samples was 64 ng/mL. Damage yields in the plasmid assay were affected by the scavenging capacity of the prepared buffer. The 0.25 mM Tris-Cl buffer showed a weak scavenging capacity. It was used to perform irradiation with MV X-rays within a few hours in the hospital [18,22].

### 2.2. Irradiation Conditions

**Figure 2:**
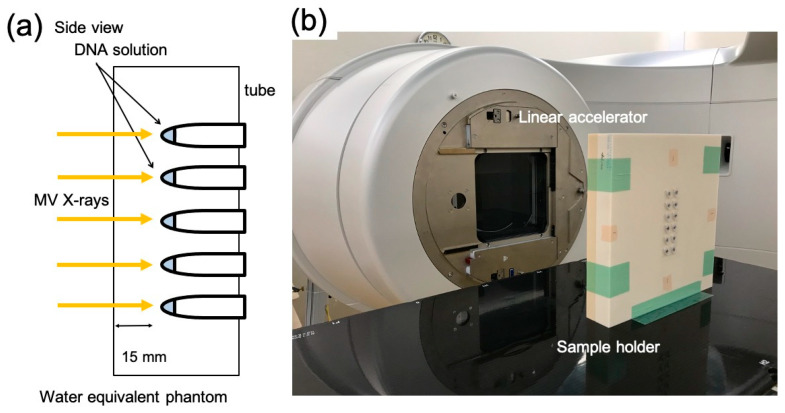
X-ray irradiation setup. (**a**) Schematic illustration and (**b**) image of the plastic phantom for the sample holder and X-ray source (gantry of linear accelerator; Linac).

**Figure 3:**
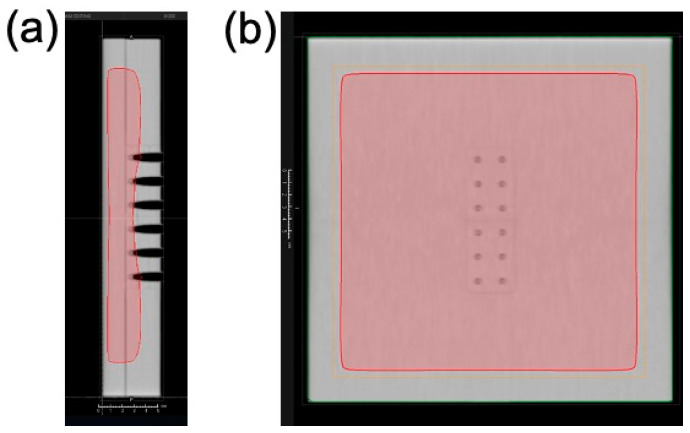
Dose distribution around the sample tube irradiated with 6 MV X-rays. Dose distribution (4 Gy) is presented in the color wash (red) from the side view (**a**) and the front view (**b**). Dose distribution was calculated using the treatment planning system.

The sample tubes containing DNA solution were placed in a holder custom-made from water-equivalent plastics (Tough Water Phantom, Kyoto Kagaku, Kyoto, Japan) for uniform dose delivery (Figure 2). The samples were irradiated with 6 MV X-rays from the side through the bottom of the tubes under aerobic conditions. The X-ray source used was Linac (TruBeam, Varian, Palo Alto, CA, USA) at Aichi Cancer Center Hospital. The gantry of Linac was rotated at 270° to irradiate the samples from the sides. The dose rate was fixed at 600 MU/min. Each irradiation dose was 4 Gy, and samples reaching the desired dose (4–20 Gy) were removed from the holder. Dose delivery was planned with a treatment planning system (Eclipse, Varian). Doses were calibrated by following the standard protocol [26]. The samples were stored at 4 °C until electrophoresis. Electrophoresis was performed within a few hours of irradiation. Dose distribution was calculated using the treatment planning system based on computed tomography (CT) images of the sample tubes fixed on the holder. For dose calculations, the thickness of the tube was considered. Figure 3 presents dose distribution in the irradiated sample tubes calculated using the treatment planning system. Isodose lines of 4 Gy are presented in the red color wash. Doses were uniformly delivered to the DNA sample solutions within 3% deviations.

### 2.3. DNA Damage Analysis

DNA damage was detected as changes in the form of plasmid DNA. Native, undamaged plasmid DNA forms a supercoiled (SC) structure. If radiation induces single-strand breaks (SSBs) in plasmid DNA, an open circular (OC) structure is formed. Furthermore, if radiation induces double-strand breaks (DSBs) in plasmid DNA, a linear (L) structure is formed [17,18,19,20,21,22]. These form changes can be quantified using agarose gel electrophoresis. The samples were separated in a 1% agarose gel at 25 V for 5 h in TBE buffer (44.5 mM Tris-borate with pH 8.4 and 1 mM EDTA) [22,24,25,27]. The gels were stained with ethidium bromide (1 μg/mL) for 20 h, destained with pure water to reduce the background brightness, and illuminated with UV light and captured using a high-sensitive camera (ImageQuant LAS 4010, GE Healthcare, Little Chalfont, UK). The brightness of each DNA band (SC, OC, L) was quantified using software (ImageQuant TL, GE Healthcare, Chicago, IL, USA). The brightness of the SC band was compensated by multiplying with a factor of 1.42 [28]. The SC and L DNA bands were quantified according to the intensity of each DNA band and normalized to the total amount of DNA. Two SSBs within a proximity of approximately 6 base pairs were detected in the L band [29].

SSB and DSB yields were calculated as previously described [22,24,25]. The SSB yield was calculated as the slope of the logarithmic plot of the fraction of SC plasmids as a function of dose. The DSB yield was calculated as the slope of the plot of the fraction of L plasmids as a function of dose. Both slopes were obtained by linear fitting using Origin software (OriginLab, Northampton, MA, USA). The SSB and DSB yields were derived by dividing each slope by the DNA mass (650 g mol^−1^ bp^−1^ × number of bp in the plasmid). The SSB and DSB yields are expressed as per DNA mass (Da) and per radiation dose (Gy), where Da is equivalent to the atomic mass unit. The DEF, a measure of the radiation sensitization effect, was calculated as the ratio of damage yield between the tested AuNP solution and the control (without AuNPs) [11,12,18,22].

Statistical analysis was performed using Tukey–Kramer test. Values were considered statistically significant at *p* < 0.05.

### 2.4. Measurement of ROS Yields

ROS yields were measured using a fluorophore whose intensity was sensitive to ROS yields [6,22]. Samples with 10 μM 2-(6-(4-amino) phenoxy-3H-xanthen-3-on-9-yl) benzoic acid (APF) (Sekisui Medical, Tokyo, Japan) and without DNA were prepared and irradiated, following the same procedure as in the plasmid assays. APF is sensitive to hydroxyl radicals and has an excitation wavelength of 490 nm and an emission wavelength of 515 nm. Aliquots of the irradiated samples were placed in a plate reader (Synergy HTX, BioTek Instruments, Winooski, VT, USA) to measure their fluorescence intensities. Quenching of APF by AuNPs was not considered, because the concentration of APF was considerably higher than that of AuNPs in the assays.

## 3. Results and Discussion

### 3.1. Characterization of AuNPs

**Figure 4:**
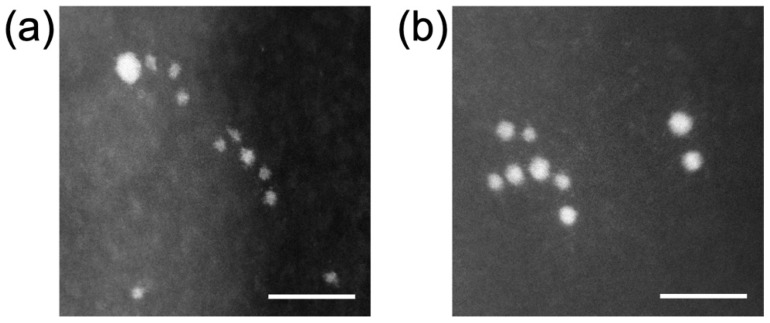
Scanning transmission electron microscopy (STEM) images of AuNPs: (**a**) 1.4 nm +AuNPs; (**b**) 2.0 nm −AuNPs. Scale bar = 10 nm.

Figure 4 shows the STEM image of +AuNPs and −AuNPs. The AuNPs were of size approximately 1–2 nm. Both AuNPs showed monodispersity and were not aggregated. The measured diameters (mean ± SD) were 1.4 ± 0.4 nm for +AuNPs and 2.2 ± 0.5 nm for −AuNPs. The zeta potentials were +14.9 mV for +AuNPs and −34.1 mV for −AuNPs, reflecting the surface charge of AuNPs.

### 3.2. Increase in DNA Damage by +AuNPs

**Figure 5:**
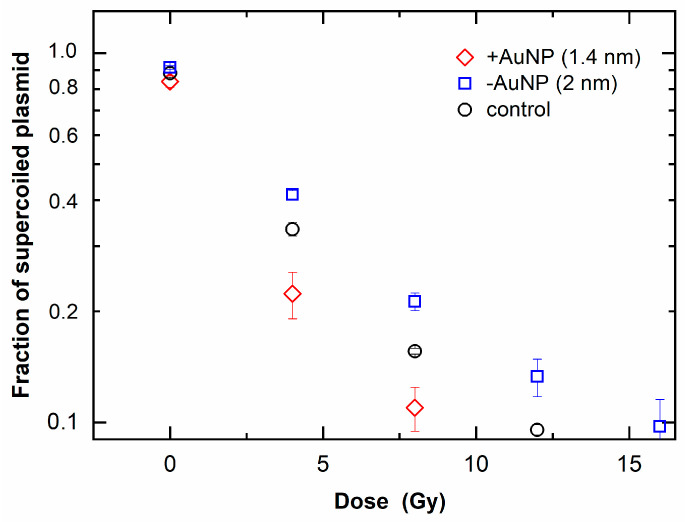
Loss of supercoiled plasmid as a function of radiation dose of 6 MV X-rays in the presence of gold nanoparticles (AuNPs).

Figure 5 shows that the fraction of SC DNA decreased with an increase in radiation dose, indicating an increase in SSBs. SC DNA was further decreased in the 1.4 nm +AuNP sample compared with that in the control sample (without AuNPs). In contrast, other samples containing −AuNPs showed no significant difference compared with the control. These findings suggest that only 1.4 nm +AuNPs promoted SSBs, thus showing radiosensitization effects.

**Figure 6:**
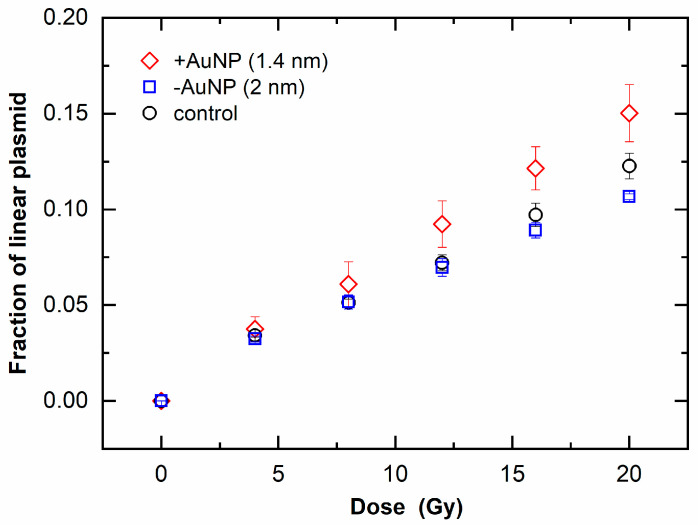
Increase in linear plasmids as a function of radiation dose of 6 MV X-rays in the presence of gold nanoparticles (AuNPs).

Figure 6 shows the fraction of linear plasmid against the irradiated dose, which indicates an increase in DSBs. When the fraction of linear plasmid increased, the response to the dose increased in the sample with 1.4 nm +AuNPs compared with that of the control, whereas the sample with −AuNPs did not significantly differ from that of the control. These findings indicate that only +AuNPs promoted an increase in DSBs, thus showing radiosensitization for DSBs.

DNA damage induced by 6 MV X-rays was increased in the presence of 1.4 nm +AuNPs. The DEF was 1.4 ± 0.2 for SSB and 1.2 ± 0.1 for DSB (Table 1), and these values were consistent with the results of cell survival assays using 6 MV X-rays. The DEF was 1.29 for lung cancer cells treated with 1.9 nm AuNPs [11], 1.17 for ovarian cancer cells treated with 50 nm AuNPs [12], and 1.36 for prostate cancer cells treated with Au nanorods [14].

The MV X-ray values were comparable to those obtained for kV X-rays in the plasmid DNA assays. The DEF values obtained with +AuNPs are consistent with previously reported values obtained with −AuNPs [17,18,19,20]. A previous study using the same buffer (0.25 mM Tris) revealed that the DEF was ~1.5 for SSBs using 160 kVp X-rays with 11.9 nm AuNPs (13 μg/mL) [18]. Our values are comparable to those obtained with Tris-EDTA buffer, which showed a higher scavenging capacity than the buffer used in this study [17].

The DEF for MV X-rays was comparable to that for 192Ir γ-rays (mean 355 keV) with 1.4 nm +AuNPs [22]. A previous study using 5 nm +AuNPs reported that the DEF was ~2.1 at 1 Gy for SSBs using 100 keV X-rays [21]. Our DEF values were lower than those obtained in 100 keV X-ray analysis. These results are consistent with the fact that lower-energy X-rays provide greater dose enhancement by interacting with AuNPs through photoelectric absorption than high-energy X-rays.

### 3.3. Effects of Surface Charge of AuNPs

Our results indicate that radiosensitization for MV X-rays occurs at a low concentration of +AuNPs. Thus, using +AuNPs in combination with radiation therapy may be useful for reducing the AuNP concentration. The concentrations of AuNPs used in our assays were approximately 200–3000-fold lower than those in previous studies using the plasmid DNA assay [17,18,19,20,21]. The AuNP concentration was 7-fold lower than that of the assumptions based on MC calculations [10].

**Figure 7:**
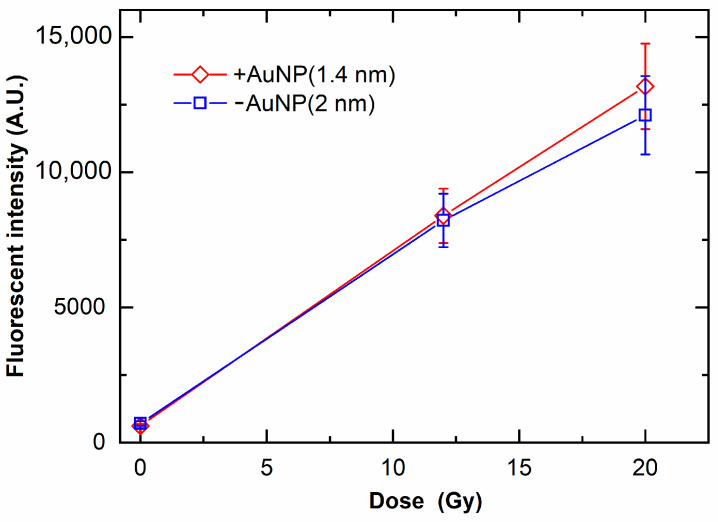
Yields of reactive oxygen species (ROS) as a function of radiation dose of 6 MV X-rays in the presence of gold nanoparticles. ROS yields are evaluated as the fluorescence intensity of the fluorescent probe sensitive to ROS yields.

Figure 7 shows the ROS yields by irradiation with MV X-rays. The ROS yields increased with the increase in radiation doses. In contrast to DNA damage, the ROS yield in the presence of +AuNPs and −AuNPs did not significantly differ. These findings indicate that the ROS yields were similar for samples with −AuNPs and +AuNPs.

Although +AuNPs increased plasmid DNA damage, −AuNPs did not significantly increase DNA damage. In contrast, the ROS yields in samples with −AuNPs and +AuNPs were similar (Figure 7). Thus, the ROS yields did not change depending on the surface charge of AuNPs. In contrast, the yields of DNA damage varied depending on the surface charge of AuNPs, possibly because +AuNPs bind to negatively charged DNA or increase the local concentration around the DNA (Figure 1).

**Figure 8:**
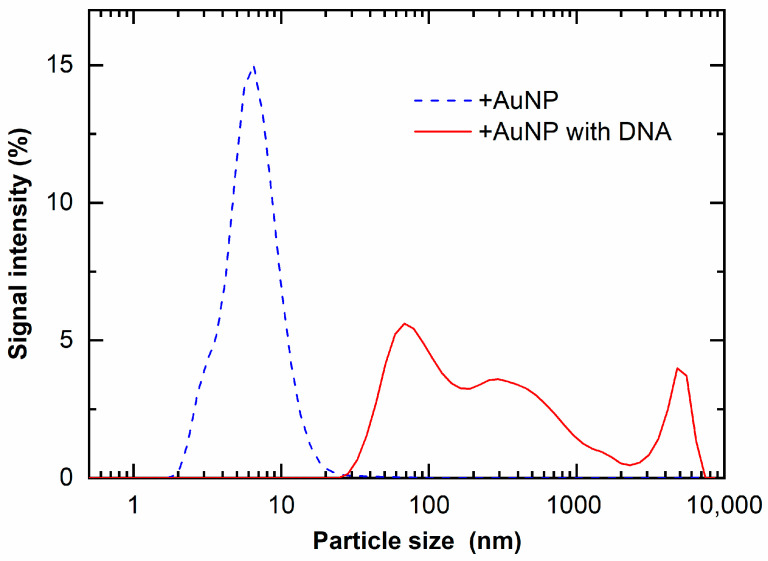
Light-scattered intensity of +AuNP solution with and without DNA.

These findings were confirmed with DLS measurements. Figure 8 shows the particle size of +AuNPs with and/without DNA measured by DLS. The apparent diameters of +AuNPs increased in the presence of DNA. These results indicate that the aggregation of +AuNPs occurred in the presence of DNA. The apparent diameters of +AuNPs were presumably increased by the binding of +AuNPs to DNA. These diameter changes were not observed for −AuNPs with/without DNA. The particle sizes measured with DLS were slightly different from those measured from STEM images. This might be because the particle size of AuNPs used in this study was close to the detection size limit of DLS, approximately 1 nm. In addition, the particle size measured by DLS is hydrodynamic diameter, not physical diameter measured from STEM images.

### 3.4. Radiosensitization Mechanism for MV X-rays

Our results indicate that the radiosensitization effects of +AuNPs for MV X-rays occurred at the DNA level; hence, we eliminated the contributions from biological effects in this study, such as cell cycle regulation and apoptosis [5]. Therefore, the radiosensitization effects were not biological effects, but rather involved other effects. One plausible explanation is that the therapeutic MV X-rays are poly-energetic, and the low-energy X-rays included in the MV X-rays induce radiosensitization through photoelectric effects [4]. In addition, Compton electrons produce lower-energy photons that trigger photoelectric effects.

The effects may have occurred through a chemical mechanism, such as ROS generation and amplification on AuNP surfaces, which are specific properties of NPs, not observed in bulk materials. Sicard-Rosselli proposed a mechanism for hydroxyl radical production in irradiated AuNP solutions [30]. Water radiolysis products generated via radiation showed a catalysis-like reaction at the water–NP interface. The mechanism of radiosensitization for MV X-rays is unclear. A detailed analysis of ROS generation by AuNPs may provide insight into the mechanism of radiosensitization by AuNPs for MV X-rays.

Combining +AuNPs with MV X-rays in radiation therapy may be useful for improving clinical outcomes. However, several issues must be resolved before +AuNPs can be clinically applied. Radiosensitization effects at a low concentration of +AuNPs should be determined at the cellular level. Another issue is the drug delivery by +AuNPs to the target site [5,31]. Interestingly, +AuNPs showed higher adsorption on the cell membrane (negatively charged) and higher (5–10-fold) internalization in the cell compared to −AuNPs, and they directly pass through the cell membrane without the endocytosis pathway [32]. Further assessment of the effect of +AuNPs on cancer cells would enable their wider application for radiation therapy in combination with MV X-rays.

## 4. Conclusions

We investigated the ability of +AuNPs to increase plasmid DNA damage induced by MV X-rays. +AuNPs can increase radiosensitization for both SSBs and DSBs at concentrations lower than those used in previous studies. +AuNP showed the radiosensitization effect for MV X-rays that have energies approximately 20 times higher than ^192^Ir source gamma-rays used in the previous work. The radiosensitization effects of +AuNPs for MV X-rays occurred at the DNA level without affecting biological pathways. −AuNPs generated a similar amount of ROS but did not show significant radiosensitization in DNA damage. Thus, dose enhancement by low concentrations of +AuNPs presumably occurs by targeting DNA.

According to the findings obtained in the present study, it is expected that the use of +AuNPs will be effectively applied to the external beam therapy using MV X-rays in addition to brachytherapy techniques using gamma-ray sources. Future assessments using cancer cells with +AuNPs would enable their wider applications to a variety of radiation therapy.

## Figures and Tables

**Table 1:** Chemical yields of DNA strand breaks and dose enhancement factors in MV X-ray irradiated samples. Data are presented as mean ± standard deviations from three independent experiments.

Experimental Conditions	Yield (breaks per Da per Gy) *		Dose Enhancement Factor **	
	Single-Strand Breaks	Double-Strand Breaks	Single-Strand Breaks	Double-Strand Breaks
1.4 nm +AuNP	(9.7 ± 1.3) × 10^−8^	(2.7 ± 0.1) × 10^−9^	1.4 ± 0.2	1.2 ± 0.1
2 nm −AuNP	(6.4 ± 0.4) × 10^−8^	(2.0 ± 0.1) × 10^−9^	0.9 ± 0.1	0.9 ± 0.1
Control	(6.8 ± 0.7) × 10^−8^	(2.2 ± 0.1) × 10^−9^	1.0	1.0

* Da (Dalton) is the unit for the molecular weight of DNA, equivalent to atomic mass units. ** Dose enhancement factor = SSB or DSB yield with treatment/yield of control.
